# Teriflunomide treatment outcomes in multiple sclerosis: A Portuguese real-life experience

**DOI:** 10.1177/23982128231185290

**Published:** 2023-07-21

**Authors:** Carla Cecília Nunes, Pedro Abreu, Filipe Correia, Irene Mendes, Ana Martins da Silva

**Affiliations:** 1 Centro Hospitalar e Universitário de Coimbra (CHUC), Coimbra, Portugal; 2 Departamento de Neurociências Clínicas e Saúde Mental, Faculdade de Medicina, Universidade do Porto, Porto, Portugal; 3 Centro Hospitalar Universitário de São João (CHUSJ), Porto, Portugal; 4 Unidade Local de Saúde de Matosinhos (ULSM) – Hospital Pedro Hispano, Matosinhos, Portugal; 5 Hospital Garcia de Orta, Almada, Portugal; 6 Centro Hospitalar Universitário de Santo António (CHUdSA), Porto, Portugal

**Keywords:** Relapsing–remitting multiple sclerosis, teriflunomide, real-world evidence, patient-reported outcomes, anxiety, satisfaction, compliance, persistence

## Abstract

Teriflunomide is an oral disease-modifying therapy for relapsing–remitting multiple sclerosis patients. A decline in physical and cognitive functions, which negatively impacts their quality of life (QoL), is observed in relapsing–remitting multiple sclerosis patients. The aim of this study was to characterise adult Portuguese relapsing–remitting multiple sclerosis patients treated with teriflunomide in routine clinical practice concerning their quality of life, comorbidities, treatment effectiveness, satisfaction, compliance and safety. TeriLIVE-QoL was a multicentre, non-interventional, prospective cohort study that collected demographic and clinical characteristics, patient-reported outcomes and adverse events from patients treated with teriflunomide of 14 mg over 2 years. Notably, around 18 months of this period occurred during the COVID-19 pandemic. Of the 99 participants, 25% were treatment-naïve. Annualised relapse rate and the score for the Hospital Anxiety and Depression Scale decreased after 1 (p = 0.01) and 2 years of treatment (p < 0.001), respectively. Convenience (p = 0.001), effectiveness (p = 0.002) and global satisfaction scores (p < 0.001) presented high values (up to 95.6) and continued to improve along the study. Treatment persistence was 77%, and compliance reached 82% 2 years after initiation. Three patients experienced serious adverse events. TeriLIVE-QoL provides real-world evidence of clinical effectiveness, high treatment satisfaction, consistent safety and improved psychiatric outcomes, associated with elevated treatment persistence and compliance in patients treated with teriflunomide.iance reached 82% 2 years after initiation. Three patients experienced serious adverse events.

## Introduction

Multiple sclerosis (MS) is a chronic inflammatory and neurodegenerative disease of the central nervous system representing the most common cause of non-traumatic disability in young adults. Still, it has an increasing prevalence in older age groups, with a heterogeneous disease activity (Goldman et al., 2010). Up to 85% of MS patients present the relapsing–remitting (RRMS) phenotype (Scott, 2019).

Teriflunomide (Aubagio^®^) is a once-daily oral disease-modifying therapy (DMT) indicated for RRMS treatment (Scott, 2019). This drug exerts immunomodulatory effects by selectively and reversibly inhibiting the mitochondrial enzyme dihydro-orotate dehydrogenase, required for *de novo* pyrimidine synthesis, thus blocking the proliferation of rapidly dividing lymphocytes (Bar-Or et al., 2014). A growing number of studies attested long-term effectiveness, safety, and tolerability of teriflunomide (Confavreux et al., 2012, 2014; O’Connor et al., 2011, 2016; Wolinsky et al., 2013). More recently, high treatment satisfaction, compliance, and persistence levels were reported in real-world clinical practice (Coyle et al., 2017; Kallmann et al., 2019; Vermersch et al., 2020).

The decline of physical and cognitive functions, inherent to this disease, can negatively impact patients’ quality of life (QoL) and represent a burden on their capacity to remain employed or perform daily activities, while affecting their personal relationships (Cadden and Arnett, 2015; Lysandropoulos et al., 2015). Thus, understanding and evaluating social and psychiatric parameters in real-life clinical practice should be considered for MS management, as they can help monitor disease progress, evaluate therapeutic effectiveness, and make individualised treatment decisions (Hobart et al., 2004; Lysandropoulos et al., 2015; Mitchell et al., 2005; Schaffler et al., 2013).

Despite the numerous studies worldwide reporting real-world evidence of teriflunomide treatment on RRMS patients (Coyle et al., 2017; Kallmann et al., 2019; Landete et al., 2021; O’Connor et al., 2016; Papp et al., 2021; Vermersch et al., 2020), none of them refers to the Portuguese population. Thus, this study (TERIFL08618) aimed to characterise a Portuguese cohort of RRMS patients under teriflunomide treatment in routine clinical practice in terms of their QoL, presence of different psychiatric comorbidities, and treatment effectiveness, satisfaction, compliance, and safety.

## Materials and methods

### Study design

TeriLIVE-QoL was a multicentre, non-interventional, prospective cohort study conducted between July 2018 and December 2021. A total of 16 central hospitals from mainland Portugal participated in this study (*Centro Hospitalar Universitário de Santo António, Centro Hospitalar Universitário de São João, Unidade Local de Saúde de Matosinhos, Hospital da Senhora da Oliveira – Guimarães, Centro Hospitalar Universitário Cova da Beira, Hospital Garcia de Orta, Centro Hospitalar de Setúbal, Centro Hospitalar Universitário de Lisboa Central, Centro Hospitalar Universitário do Algarve – Portimão, Centro Hospitalar Universitário do Algarve – Faro, Centro Hospitalar Universitário de Lisboa Norte, Centro Hospitalar de Lisboa Ocidental, Centro Hospitalar e Universitário de Coimbra, Centro Hospitalar de Entre o Douro e Vouga, Centro Hospitalar do Tâmega e Sousa*, and *Centro Hospitalar Barreiro Montijo*), and the study participants were recruited in accordance to routine clinical practice.

### Study population

A sample size of 95 patients was determined based on the estimated prevalence of MS in Portuguese adults, the proportion of RRMS patients treated with teriflunomide, and the observed maximum standard deviation of the Multiple Sclerosis Impact Scale (MSIS)-29 components in community samples.

Patients who fulfilled the inclusion criteria were eligible for the study: adult outpatients (⩾ 18 years old), diagnosed with RRMS, who started teriflunomide treatment in the 4 weeks before enrolment, had no record of an acute episode during the 30 days prior to the study entry, had an Expanded Disability Status Scale (EDSS) score ⩽5.5, were able to completing the patient-reported outcomes (PROs) questionnaires, and had signed written informed consent.

Whenever patients met at least one exclusion criteria, they were not included in the study: patients who did not complete the PROs, were participating in other interventional studies that interfered with the current study, and were concomitantly using an investigational drug.

### Study objectives

The primary objective of TeriLIVE-QoL was to characterise a Portuguese cohort of RRMS patients under treatment with teriflunomide 14 mg in routine clinical practice, over a 2-year period, using PROs. For that, patients’ QoL, through the MSIS-29 (Hobart et al., 2001), was evaluated, and the effect of teriflunomide on psychiatric comorbidities was determined, namely depression and anxiety. This is a 29-item self-report measure, which is scored by summing the responses across items and then converted to a 0–100 scale, where 100 indicates a greater impact of disease on daily function. Fatigue was measured by the Modified Fatigue Impact Scale-5 item version (MFIS-5) (Guidelines, 1998) and depression and anxiety by the Hospital Anxiety and Depression Scale (HADS) (Zigmond and Snaith, 1983). MFIS-5 is a self-assessment tool for measuring the impact of fatigue on cognitive, physical, and psychosocial function that ranges from 0 to 20, with higher scores indicating more severe fatigue (Meca-Lallana et al., 2019). HADS is a 14-item scale with 7 items each for anxiety and depression. Scoring for each item ranges from 0 to 3 and a subscale score > 8 denotes anxiety or depression (Rishi et al., 2017). The effectiveness of teriflunomide in delaying physical disability progression and reducing the annualised relapse rate (ARR) was also evaluated. Disability progression was measured using the EDSS (Kurtzke, 1955) and the Patient Determined Disease Steps (PDDS) (Shear et al., 1997), taking as reference scores obtained at the time of teriflunomide initiation. ARR in the first and second years after treatment initiation was compared with the ARR in the pre-treatment year. Furthermore, this study aimed to evaluate patients’ treatment satisfaction using the four domains of the Treatment Satisfaction Questionnaire for Medication (TSQM 1.4; global satisfaction, convenience, side effects, and effectiveness) (Atkinson et al., 2004), their working capacity and daily activity using the Work Productivity and Activity Impairment Questionnaire (WPAI) (Reilly et al., 1993), treatment persistence and compliance, and teriflunomide safety, based on the report of adverse events (AEs). Persistence on teriflunomide was defined as the time between treatment initiation and discontinuation. When there was no discontinuation, the date when the patient quit or withdrew from the study was considered. Adherence was measured as the frequency of patients who took all prescribed teriflunomide doses.

### Data collection

Data corresponding to a 2-year follow-up were prospectively collected. Of note, around 18 months of the study period occurred during the COVID-19 pandemic. By the time of study enrolment (baseline), the main data collected were demographic data, MS disease history, DMT history, presence of comorbidities, and PROs (MFIS-5, HADS, EDSS, PDDS, MSIS-29, TSQM, and WPAI). Information on MS disease history and DMT history was collected retrospectively, without any specific time interval. The occurrence of relapses was the only variable collected in the 12 months prior to the patients’ enrolment in the study and the start of teriflunomide. In the subsequent visits (6, 12, and 24 months), data collected were PROs, clinical data, treatment compliance, and presence of AEs. In naïve patients, only data collected 6 months after study initiation were considered when evaluating TSQM and treatment persistence and compliance.

### Statistical analysis

Continuous variables were summarised considering mean and standard deviation. For categorical variables, absolute and relative frequencies were used. There was no imputation of missing data.

Normality distribution of continuous variables was examined using histograms, Q–Q plots, and the Shapiro–Wilk test. In addition, a comparison of paired observations between the baseline and each follow-up visit was performed considering the non-parametric Wilcoxon test for paired samples. For scales with more than one dimension, the significance level was first adjusted for the number of dimensions and then for the number of multiple comparisons, using Bonferroni’s method. Clinical significance was assessed using effect size (ES), which was calculated by dividing the mean change at baseline by the SD of the change and defined according to the limits set out by Cohen: < 0.2, negligible; 0.2–0.5, small; 0.5–0.8, moderate; > 0.8, high (Cohen, 1988).

Kaplan–Meier estimator was used to assess teriflunomide persistence and the 95% confidence interval (CI) for those estimates at 6, 12, and 24 months. The persistence time was defined as the time from teriflunomide initiation until its discontinuation or last medical contact. Univariable and multivariable Cox regression models were adjusted to determine predictors of drug discontinuation.

A two-sided p-value of less than 0.05 was considered statistically significant in all analyses. Statistical analysis was performed using the R software version 4.1.2 (Vienna, Austria).

### Ethics

TeriLIVE-QoL was conducted in accordance with the International Conference on Harmonisation Guidelines for Good Clinical Practice and the Declaration of Helsinki, and was approved by the local ethical committee (Study No. TERIFL08618). All participants gave written informed consent before entering the study.

## Results

### Demographics and clinical characterisation of RRMS patients at baseline

A total of 99 patients completed the baseline assessment. Detailed information regarding demographics and MS history is provided in Table 1. The data were collected from a total of 99, 96, 85, and 72 patients at baseline, 6, 12, and 24 months, respectively.

**Table 1.** Demographic and clinical characteristics of patients at baseline.

Demographic characteristics	n = 99
Age (years)	
M ± SD	47 ± 11
[Min, Max]	[23, 76]
Female, n (%)	68 (68.7)
Marital status, n (%)	
Married	60 (60.6)
Single	21 (21.2)
Divorced/separated	15 (15.2)
Widower	2 (2.0)
Did not answer	1 (1.0)
Education, n (%)	
High school	44 (44.4)
University	29 (29.3)
Primary school	24 (24.2)
Did not answer	1 (1.0)
Smoking status, n (%)	
Never	50 (50.5)
Former	25 (25.3)
Current	24 (24.2)
Clinical characteristics	n = 99
Time between disease onset and patient diagnosis (years), M ± SD	2.6 ± 5.1
Time since patients’ last MS relapse (years), M ± SD	4.5 ± 5.1
Comorbidities, n (%)	74 (74.7)
Depression	26 (26.3)
Hyperlipidaemia	22 (22.2)
Fatigue	20 (20.2)
Hypertension	19 (19.2)
Hypovitaminosis D	15 (15.2)
Anxiety	14 (14.1)
Symptomatic therapy for MS, n (%)	42 (42.4)
Previous DMT, n (%)	75 (75.8)
Injectable	64 (85.3)
Interferon β-1a	28 (37.3)
Glatiramer acetate	24 (32.0)
Peginterferon β-1a	7 (9.3)
Interferon β-1b	5 (6.7)
Oral	10 (13.3)
Dimethyl fumarate	9 (12.0)
Fingolimod	1 (1.3)
Infusion	1 (1.3)
Natalizumab	1 (1.3)
DMT treatment duration (months), M ± SD	63.0 ± 63.3
Time since DMT discontinuation (days), M ± SD	125.6 ± 320.0
⩽180 days	65 (86.7)
>180 days	10 (13.3)
ARR over the previous 12 months, M ± SD	0.24 ± 0.47
EDSS score, M ± SD	1.57 ± 1.31

MS: multiple sclerosis; ARR: annualised relapse rate; DMT: disease-modifying therapy; EDSS: expanded disability status scale; MS: multiple sclerosis.

At inclusion, patients’ mean age was 47.0 ± 11 years, ranging from 23 to 76, and 68.7% of them were females. Most patients (74.7%) had comorbidities at baseline, among which depression (26.3%), hyperlipidaemia (22.2%), fatigue (20.2%), hypertension (19.2%), and anxiety (14.1%) were the most common. On average, patients had their last MS relapse 4.5 ± 5.1 years before starting teriflunomide.

Approximately 25% of patients were DMT-naïve, whereas the other 75% switched to teriflunomide from a previous treatment. From now on, the latter will be referred to as ‘switchers’. The last prescribed DMT was mostly injectable (85.3%). Patients discontinued their previous treatment mainly due to side effects (82.7%) and therapeutic convenience (10.7%), that is, medication regimen related to the complexity and frequency of dosing. Demographic and clinical characteristics of DMT-naïve patients and switchers are detailed in Table S1. Briefly, although both groups had similar characteristics, there was a higher proportion of female patients who switched from a previous DMT. Also, switchers experienced their last MS relapse longer than DMT-naïve patients.

The mean ARR in the year before study initiation was 0.24 (0.67 in DMT-naïve patients, 0.11 in switchers), with 77.8% of patients recording no relapse during this period. The overall population had a mean EDSS of 1.57 (1.85 in DMT-naïve patients, 1.47 in switchers), with 79.8% of patients having an EDSS ⩽ 2.

### Effectiveness

Disability was evaluated using the EDSS and PDDS scales (ranging 0–10 and 0–6, respectively), whose scores remained stable during the study period. Mean EDSS at study entry, Month 12, and Month 24 were 1.57, 1.75 (p = 0.682), and 1.61 (p = 0.887), respectively (Figure S1A). Concerning PDDS, the mean values were 2.1 at baseline, 12 months (p = 0.959), and 24 months (p = 0.507) follow-up visits (Figure S1B).

**Figure 1.**
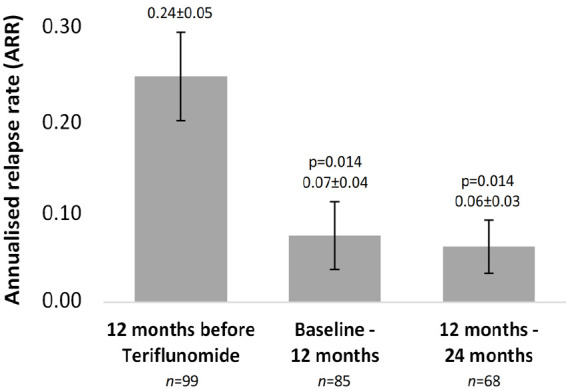
Mean (±SEM) ARR of patients 12 months before and 24 months after teriflunomide initiation. Multiple comparisons were performed using the Wilcoxon test for paired samples and the p-value was adjusted by Bonferroni’s method: 0.050/2 = 0.025.

The mean ARR significantly reduced from 0.24 12 months before teriflunomide initiation to 0.07 after the first year of treatment (p = 0.01) (Figure 1). This reduction was maintained until the end of the study (0.06, p = 0.014), in which the frequency of patients free from relapses reached 94.1%. DMT-naïve patients, who had higher mean ARR values in the year before teriflunomide initiation compared to switchers, experienced a statistically significant reduction in ARR 12 months after starting teriflunomide (p = 0.021). In contrast, switchers maintained stable ARR values over the period analysed (p = 0.240) (Figure S2).

### QoL

Global MSIS-29 scores for QoL remained stable from baseline to the end of this study (physical: 21.8 versus 24.2, p = 0.836; psychological: 30.2 versus 28.3, p = 0.142; global: 24.4 versus 25.4, p = 0.604) (Figure S2).

### Fatigue, depression, and anxiety

**Figure 2.**
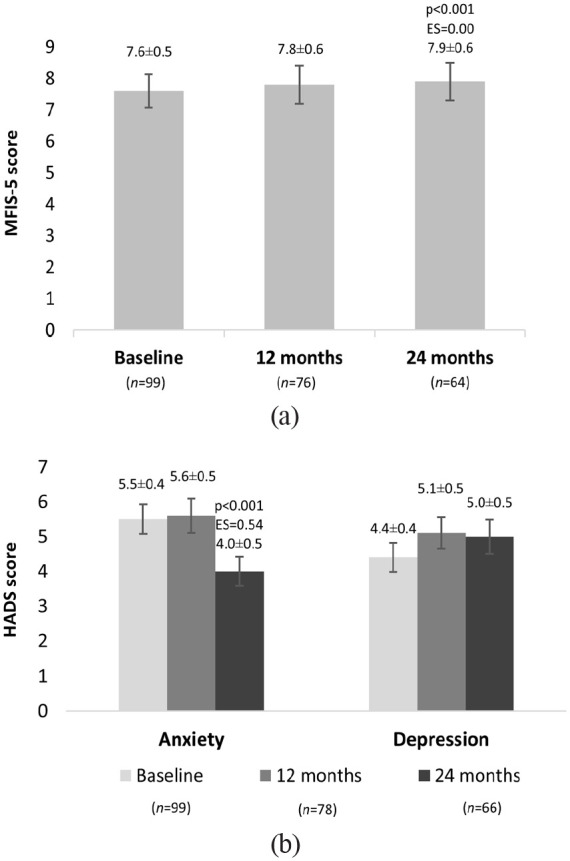
Mean (±SEM) of (a) fatigue and (b) anxiety and depression scores of RRMS patients at baseline and after 12- and 24-month follow-ups, as measured by the MFIS-5 and HADS scales. Multiple comparisons were performed between baseline and each follow-up visit using the Wilcoxon test for paired samples and the p-value was adjusted by Bonferroni’s method: 0.050/2 = 0.025 for MFIS-5 and 0.025/4 = 0.006 for HADS.

MFIS-5 mean values increased after a 24-month period, when compared to baseline (7.6 versus 7.9, p < 0.001) (Figure 2(a)). However, the clinical significance of this change was negligible, according to Cohen’s criteria (ES = 0.00). At the 12-month follow-up visit, there was no significant alteration in fatigue levels (7.8, p = 0.357).

Concerning the HADS scale, while patients reported no change in depression over time (baseline: 4.4; 12 months: 5.1, p = 0.163; 24 months: 5.0, p = 0.323), mean anxiety scores significantly decreased at Month 24 (baseline: 5.5; 12 months: 5.6, p = 0.357; 24 months: 4.0, p < 0.001), with moderate ES values for the change (ES = 0.54). In addition, at the 24-month visit, depression registered a maximum value of 18.0, whereas 20.0 was the highest reported value for anxiety at baseline (Figure 2(b)).

### Treatment satisfaction

Teriflunomide treatment satisfaction was evaluated separately in naïve and patients switching from previous DMT. Overall, high mean scores were observed, mainly in the convenience and global satisfaction domains (up to 95.6), indicating higher treatment satisfaction, that is, the degree to which patients perceive that the treatment fulfils their health needs.

**Figure 3.**
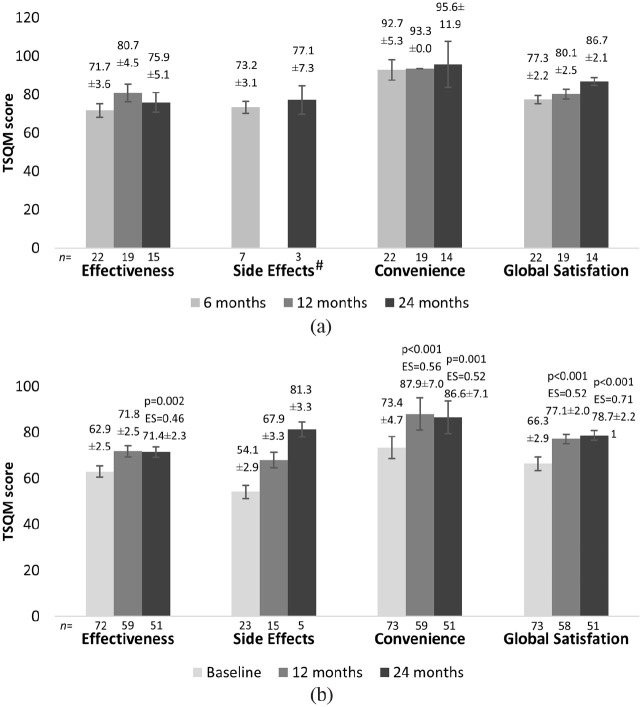
Mean (±SEM) TSQM effectiveness, side effects, convenience, and global satisfaction scores for the evaluation of treatment satisfaction in (a) DMT-naïve and (b) patients who switched from previous DMT. Multiple comparisons were performed using the Wilcoxon test for paired samples and the p-value was adjusted by Bonferroni’s method: 0.013/2 = 0.0065. ^#^Missing bar due to n = 2.

Treatment satisfaction in DMT-naïve patients was assessed at 6, 12, and 24 months after teriflunomide initiation, with no significant alterations being registered at any timepoint, when compared to the 6-month period (effectiveness: 12 months (p = 0.009), 24 months (p = 0.270); convenience: 12 months (p = 0.448), 24 months (p = 0.611); global satisfaction: 12 months (p = 0.387), 24 months (p = 0.261)) (Figure 3(a)). Few patients reported side effects, and these were only possible to assess at Months 6 and 24. Notably, treatment satisfaction among DMT-naïve patients was high at all timepoints (ranging from 71.7 to 95.6).

In patients who switched from the previous DMT, treatment satisfaction was evaluated at study entry and after 12 and 24 months (Figure 3(b)). However, 12 months after teriflunomide initiation, convenience (73.4 versus 87.9; p < 0.001, ES = 0.56) and global satisfaction (66.3 versus 77.1; p < 0.001, ES = 0.52) scores significantly increased, when compared to baseline, with moderate ES values. These results were maintained until the end of the study (convenience: 86.6, p = 0.001, ES = 0.52; global satisfaction: 78.7, p < 0.001, ES = 0.71). Effectiveness scores remained stable for 12 months (p = 0.012) and significantly increased between baseline and the end of the study (62.9 versus 71.4; p = 0.002, ES = 0.46). Mean side effects scores showed no significant alterations at any timepoint (12 months: p = 0.269; 24 months: p = 0.198).

### WPAI

The impact of MS on work productivity and daily activities of RRMS patients was evaluated using the WPAI questionnaire (Table 2). For those employed in the 7 days prior to the follow-up visit, no differences were observed in terms of work time missed (p = 0.037), impairment while working (p = 0.531), and overall work impairment (p = 0.970) between baseline and the last visit. Similarly, no differences were observed in activity impairments due to health (p = 0.117).

**Table 2.** WPAI scores of patients employed in the past 7 days at each follow-up visit.

WPAI	Baseline	12 months	24 months
Employed in the last 7 days	n = 99	n = 78	n = 66
No	45 (45.5%)	37 (47.4%)	35 (53.0%)
Yes	54 (54.5%)	41 (52.6%)	31 (47.0%)
If employed, M ± SD	n = 50^a^	n = 35^a^	n = 29^a^
% Work time missed due to health problems	7.8 ± 23.8	12.0 ± 28.6	1.1 ± 3.6
% Impairment while working due to health problems	12.1 ± 17.3	15.4 ± 19.5	14.8 ± 23.4
% Overall work impairment due to health problems	13.7 ± 18.1	26.8 ± 30.5	15.5 ± 24.2
All patients, in the last 7 days	n = 99	n = 78	n = 66
% Activity impairment due to health problems	26.7 ± 29.2	24.1 ± 26.2	23.6 ± 25.4

WPAI: Work Productivity and Activity Impairment Questionnaire.

Multiple comparisons were performed using the Wilcoxon test for paired samples and the p-value was adjusted by Bonferroni’s method: 0.017/2 = 0.0085.

a The observed difference in the number of patients is explained by the fact that some of them did not answer all the questions.

Although this study did not evaluate patients’ employment status, the data indicate that some patients alternated working periods over the 24 months of the study. In addition, four patients started working during the study period but stopped their activity within 6–18 months. Eight employed patients stopped working, of which three returned to work in 6 or 12 months. Regarding the other employed patients, we cannot assume they kept the same job between visits.

### Treatment persistence and compliance

**Figure 4.**
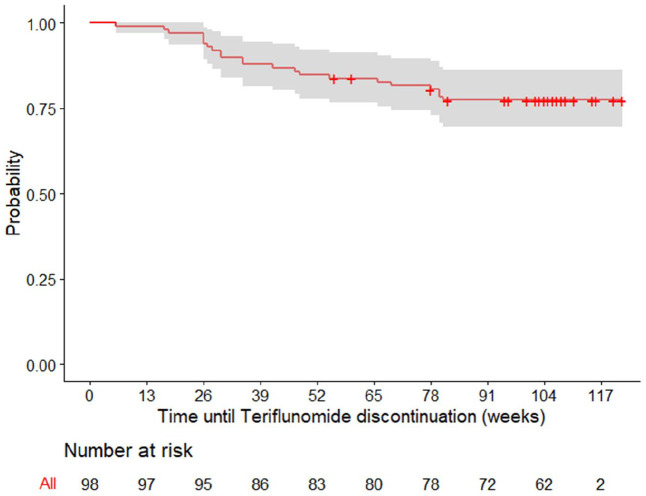
Teriflunomide drug persistence (Kaplan–Meier estimator) in relation to time.

Teriflunomide treatment persistence after 6, 12, and 24 months was 93.9% (95% CI (89.2, 98.7)), 84.7% (95% CI (77.9, 92.1)), and 77.4% (95% CI (69.5, 86.2)), respectively (Figure 4). Patients also reported high adherence, particularly 73.0% (65 patients) 6 months after study initiation and 82.0% (50 patients) by the end of the study.

Over the study period, 23 patients (23.2%) discontinued teriflunomide treatment. Even though reasons for discontinuation could not be identified in most cases, 20 patients reported AEs at the same time, namely skin and subcutaneous disorders (n = 9, 39.1%), gastrointestinal disorders (n = 5, 21.7%), and nervous system disorders (n = 4, 17.4%). Of note, time since the last relapse (< 3 years), patients’ age (< 50 years), and smoking habits (current smokers) were found to be independent predictive factors for teriflunomide discontinuation (Table S1 and Figure S3).

### Safety and AEs

A total of 156 AEs were experienced by 60 patients (60.6%), of which 16 (64.0%) were DMT-naïve and 44 (58.7%) were patients who transitioned from previous DMT (Table 3). They were mostly mild (48.1%) or moderate (45.5%) and classified as non-serious (97.4%).

**Table 3.** Adverse events classified by MedDRA system.

Adverse events	n = 156
Skin and subcutaneous tissue disorders	27 (17.3%)
Nervous system disorders	22 (14.1%)
Gastrointestinal disorders	16 (10.3%)
General disorders and administration site conditions	13 (8.3%)
Cardiac disorders	12 (7.7%)
Musculoskeletal and connective tissue disorders	11 (7.1%)
Psychiatric disorders	10 (6.4%)
Infections and infestations	8 (5.1%)
Immune system disorders	6 (3.8%)
Respiratory, thoracic, and mediastinal disorders	4 (2.6%)
Investigations	4 (2.6%)
Eye disorders	4 (2.6%)
Blood and lymphatic system disorders	3 (1.9%)
Ear and labyrinth disorders	3 (1.9%)
Metabolism and nutrition disorders	3 (1.9%)
Hepatobiliary disorders	2 (1.3%)
Neoplasms benign, malignant, and unspecified (including cysts and polyps)	2 (1.3%)
Injury, poisoning, and procedural complications	1 (0.6%)
Surgical and medical procedures	1 (0.6%)
Reproductive system and breast disorders	1 (0.6%)
Renal and urinary disorders	1 (0.6%)
Vascular disorders	1 (0.6%)
Unspecified	1 (0.6%)

The most frequently reported AEs, classified according to the MedDRA system, were skin and subcutaneous tissue disorders (17.3%), nervous system disorders (14.1%), namely paraesthesia (n = 5, 22.7%) and migraine (n = 3, 13.6%), and gastrointestinal disorders (10.3%). There were also eight (5.1%) cases of infections and infestations, of which three were due to COVID-19. Two patients (1.3%) reported hepatobiliary disorders, of which one was likely due to teriflunomide.

Serious AEs were observed in three patients (1.9%) and are depicted in Table 4. Only one death was reported during the study period due to MS-unrelated causes, in a 53-year-old male patient, who had the first MS symptoms 20 years before teriflunomide initiation.

**Table 4.** Characteristics of patients with serious AE.

Serious AE	n = 3
Surgery for ovarian mucinous neoplasm	
Age (years)	48
Gender	Female
Time between disease onset and baseline (years)	12
Time between teriflunomide initiation and the AE report (years)	1.5
Previous DMT	Yes
Causality with teriflunomide treatment	Not possible to access
Left hemibody paraesthesia and aggravated hemiparesis	
Age (years)	39
Gender	Male
Time between disease onset and baseline (years)	2
Time between teriflunomide initiation and the AE report (years)	1
Previous DMT	Yes
Causality with teriflunomide treatment	Not related
Lobular carcinoma in situ in the right breast	
Age (years)	58
Gender	Female
Time between disease onset and baseline (years)	3
Time between teriflunomide initiation and the AE report (years)	1.5
Previous DMT	No
Causality with teriflunomide treatment	Not related

AE: adverse event; DMT: disease-modifying therapy.

## Discussion

TeriLIVE-QoL was an observational study conducted in real-world clinical practice that provides insightful data regarding the use of teriflunomide 14 mg in a Portuguese population of RRMS patients. The study comprised an observation period of 2 years, further supporting previous evidence of teriflunomide effectiveness, satisfaction, compliance, and safety.

Many MS patients face a decline in physical and cognitive functions while suffering from psychiatric manifestations (Lysandropoulos et al., 2015). Here, a significant proportion of patients reported depression (27.3%), fatigue (20.2%), and anxiety (14.1%) already at the time of study initiation. After 2 years of treatment, there was a reduction in anxiety and an increase in fatigue, but with negligible clinical significance, and no changes in depression. The reduction in anxiety was an unexpected finding, especially considering that patients endured the harsh reality of the COVID-19 pandemic, a period of social isolation with severe psychological impact on any patient. Interestingly, a meta-analytic study examining the impact of the pandemic on psychological variables in MS patients revealed elevated levels of depression, anxiety, and stress compared to healthy controls. However, no significant alterations were observed within the MS patient group before and during the pandemic (Altieri et al., 2022).

The psychological variables can negatively impact the patients’ QoL. In the TeriLIVE-QoL study, there was no significant alteration in QoL, as evaluated by the MSIS-29 scale, which aligns with others (Coyle et al., 2017). This finding may be attributed to the stable levels of depression and fatigue, and decreased levels of anxiety observed throughout the study. However, considering the favourable impact on anxiety, it is reasonable to assume that if psychological and QoL assessments had been conducted at a time outside the COVID-19 pandemic, the results would likely have been even more positive.

A possible explanation for the improvement in anxiety may be related to the high treatment satisfaction observed in the study, which has previously been shown to improve clinical outcomes in RRMS patients (Haase et al., 2016). While DMT-naïve patients reported the highest TSQM scores (up to 95.6), patients switching from previous DMT showed improved convenience, effectiveness, and global satisfaction scores. This is in line with other real-world studies showing significantly improved treatment satisfaction of patients switching from a prior injectable or oral DMT to teriflunomide (Coyle et al., 2017; Kallmann et al., 2019). The observation of higher convenience scores in DMT-naïve patients, as compared to switchers, is an interesting observation, as previous studies have reported similar (Ralf et al., 2017) or lower (Kallmann et al., 2021; Miller, 2021) convenience scores in patients who have not previously received DMT. Regardless, teriflunomide has demonstrated effectiveness in treating RRMS in both patient populations (Kallmann et al., 2021; Lazzaro et al., 2022; Papp et al., 2021).

These observations are further demonstrated by high treatment persistence (77.4% by the end of the study) and adherence (up to 82.0%). Moreover, considering the pandemic period in which this study was conducted, convenience of teriflunomide may be one of the reasons why patients’ compliance remained high. In fact, oral DMTs were reported to have higher compliance and lower discontinuation rates when compared to injectable ones (Agashivala et al., 2013; Duquette et al., 2019; Vermersch et al., 2020). We can also hypothesise that the aforementioned values are due to a reasonable disease control with teriflunomide, without the need for more aggressive treatments, and from good drug tolerability.

In this study, a higher probability for teriflunomide discontinuation was associated with a relapse history in the previous 3 years, younger age, and patient’s smoking status. These are novel results that complement previously published data reporting that patients with higher EDSS at baseline were at higher risk of discontinuing oral DMTs (Ferraro et al., 2018).

The use of teriflunomide proved effective in this cohort, as EDSS and PDDS disability measures were stable, and the ARR significantly decreased in the first year of treatment. These observations further support teriflunomide’s effectiveness in the long-term (Coyle et al., 2017; O’Connor et al., 2016; Oh et al., 2021).

MS-related disabilities and comorbidities not only affect QoL but also impose a significant burden on a patient’s capacity to remain employed and perform daily activities (Cadden and Arnett, 2015). When evaluating the work capacity of those employed at the time of study visits, which corresponded to about half of the study population, there were no differences throughout the study. However, we cannot assume patients did not exchange jobs between visits due to MS-related causes, as this study did not collect employment-related information. Likewise, participants reported no alterations in their activity impairment due to MS.

Concerning the safety profile of teriflunomide, the incidence of the most frequent AEs was consistent with other studies, thus reinforcing previous evidence attesting to teriflunomide safety (Coyle et al., 2017; Kallmann et al., 2019, 2021; O’Connor et al., 2011).

Despite the difficulties inherent to the COVID-19 pandemic in the follow-up of patients, this work was successfully conducted, which may be explained by teriflunomide safety and convenience.

Several limitations are associated with this study. The first is related to the limited number of participants. Even though this is a multicentre study that geographically represents Portugal, a higher sample size would provide more robust evidence. Also, not all participants were compliant with PROs, and the number of responders was lower than expected, more evident on the TSQM scale. Also, this study did not capture employment-related information, despite its relevance for MS evaluation. Finally, the lack of a comparator and the short follow-up period may impair validity and generalisability of data and limit conclusions.

In conclusion, this study provides evidence of improved anxiety outcomes in RRMS patients treated with teriflunomide in the real world and during the pandemic period, which may be related to the high levels of treatment satisfaction, persistence, and compliance in both naïve patients and those who have switched from other DMTs. In addition, it further supports previous findings that teriflunomide is an effective and safe DMT for treating patients with mild to moderate disease activity.

## Supplemental Material

sj-docx-1-bna-10.1177_23982128231185290 – Supplemental material for Teriflunomide treatment outcomes in multiple sclerosis: A Portuguese real-life experience Click here for additional data file. Supplemental material, sj-docx-1-bna-10.1177_23982128231185290 for Teriflunomide treatment outcomes in multiple sclerosis: A Portuguese real-life experience by Carla Cecília Nunes, Pedro Abreu, Filipe Correia, Irene Mendes and Ana Martins da Silva in Brain and Neuroscience Advances
