# Supplementary material for: Teriflunomide treatment outcomes in multiple sclerosis: A Portuguese real-life experience
Source: Brain Neurosci Adv. 2023 Jul 21;7:23982128231185290. doi: 10.1177/23982128231185290 (PMC10363901; doi:10.1177/23982128231185290)
Supplement: sj-docx-1-bna-10.1177_23982128231185290 – Supplemental material for Teriflunomide treatment outcomes in multiple sclerosis: A Portuguese real-life experience [file sj-docx-1-bna-10.1177_23982128231185290.docx]

**SUPPLEMENTARY INFORMATION**

**Table S1**. **Demographic and clinical characteristics of DMT naïve patients and those who switched from a previous DMT (switchers) at baseline.**

| **Demographic characteristics** | **Naïve**  **(*n=*24)** | **Switchers**  **(*n=*75)** |
| --- | --- | --- |
| Age (years) |  |  |
| Mean±SD | 46±12 | 47±10 |
| [Min, Max] | [23, 76] | [25, 69] |
| Female, *n* (%) | 13 (54.2%) | 55 (73.3%) |
| Marital status, *n* (%) |  |  |
| Married | 16 (66.7%) | 44 (58.7%) |
| Single | 7 (29.2%) | 14 (18.7%) |
| Divorced/Separated | 1 (4.2%) | 14 (18.7%) |
| Widower | 0 (0.0%) | 2 (2.7%) |
| Did not answer | 0 (0.0%) | 1 (1.3%) |
| Education, *n* (%) |  |  |
| High school | 9 (37.5%) | 35 (46.7%) |
| University | 10 (41.7%) | 19 (25.3%) |
| Primary school | 5 (20.8%) | 19 (25.3%) |
| Did not answer | 0 (0.0%) | 2 (2.7%) |
| Smoking status, *n* (%) |  |  |
| Never | 7 (29.2%) | 43 (57.3%) |
| Former | 11 (45.8%) | 14 (18.7%) |
| Current | 6 (25.0%) | 18 (24.0%) |
| **Clinical characteristics** |  |  |
| Time between disease onset and patient diagnosis (years), mean±SD | 2.2±6.1 | 2.8±4.7 |
| Time since patients’ last MS relapse (years), mean±SD | 1.2±2.0 | 5.6±5.4 |
| Presence of comorbidities, *n* (%) | 18 (75.0%) | 56 (74.7%) |
| Depression | 1 (4.2%) | 25 (33.3%) |
| Hyperlipidaemia | 5 (20.8%) | 17 (22.7%) |
| Fatigue | 6 (25.0%) | 14 (18.7%) |
| Hypertension | 3 (12.5%) | 16 (21.3%) |
| Hypovitaminosis D | 7 (29.2%) | 8 (10.7%) |
| Anxiety | 2 (8.3%) | 12 (16.0%) |
| Symptomatic therapy for MS, *n* (%) | 8 (33.3%) | 34 (45.3%) |
| ARR over the previous 12 months, mean±SD | 0.67±0.64 | 0.11±0.31 |
| EDSS score, mean±SD | 1.85±1.49 | 1.47±1.5 |

**Table S2. Predictive factors of teriflunomide discontinuation.**

| Predictors | Beta | SE | p value | HR | 95%CI-LL | 95%CI-UL |
| --- | --- | --- | --- | --- | --- | --- |
| Last relapse ≥3 years | -0.878 | 0.416 | 0.051 | 0.416 | 0.172 | 1.004 |
| Age | -0.065 | 0.937 | 0.005 | 0.937 | 0.895 | 0.980 |
| Current smoker | 0.973 | 2.645 | 0.027 | 2.645 | 1.115 | 6.272 |

CI: confidence interval; LL: lower limit; HR: hazard ratio; SE: standard error; UL: upper limit

**A**

(*n*=64)

(*n*=77)

(*n*=99)

1.61±0.17

1.75±0.16

1.57±0.13

**B**

(*n*=66)

(*n*=78)

(*n*=99)

2.1±0.15

2.1±0.16

2.1±0.16

**Figure S1.** **Mean (±SEM) (A) EDSS and (B) PDDS score values of patients at baseline, 6-, 12-, and 24-months.** Multiple comparisons were performed considering the Wilcoxon test for paired samples and the p-value was adjusted by Bonferroni’s method: 0.050/2=0.025.

0.67±0.13

**Annualised relapse rate (ARR)**

17

51

75

22

63

24

*n*=

p=0.021

0.20±0.11

0.11±0.40

0.03±0.03

0.04±0.03

0.12±0.08

**Figure S2. Mean (±SEM) ARR of DMT naïve patients and those who switched from a previous DMT (switchers) 12 months before and in the 24 months after teriflunomide initiation.** Multiple comparisons with the 12 months before teriflunomide initiation were performed considering Wilcoxon test for paired samples and the p value was adjusted by the Bonferroni’s method: 0.050/2=0.025.

30.2±2.2

23.6±2.2

24.2±2.8

24.4±1.9

(*n*=99)

28.3±2.6

27.2±2.7

21.8±2.0

25.5±2.5

24.7±2.2

(*n*=78)

(*n*=66)

**Figure S2**. **Mean (±SEM) MSIS-29 physical, psychological, and global scores of patients.** Multiple comparisons were performed considering Wilcoxon test for paired samples and the p value was adjusted by the Bonferroni’s method: 0.017/2=0.0085.


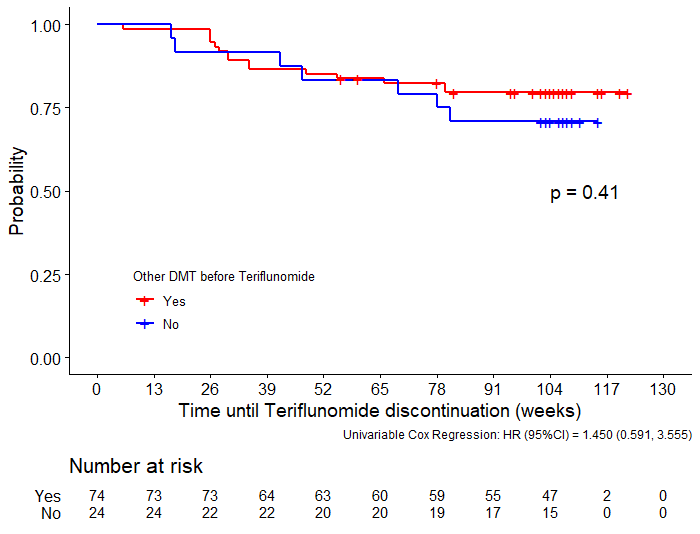

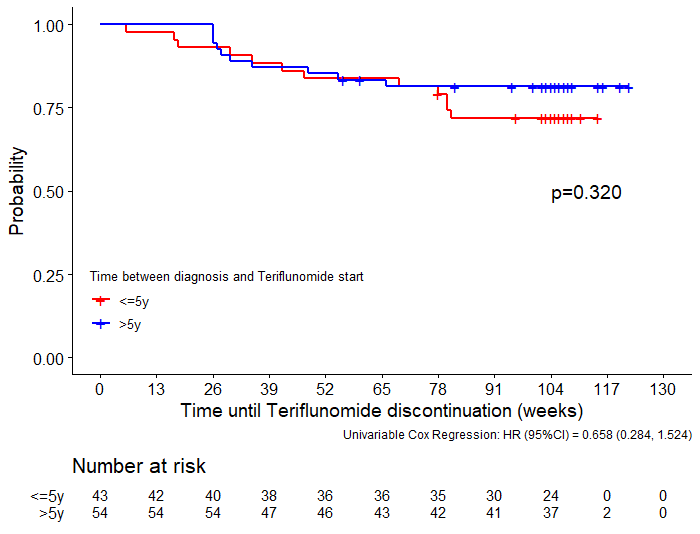

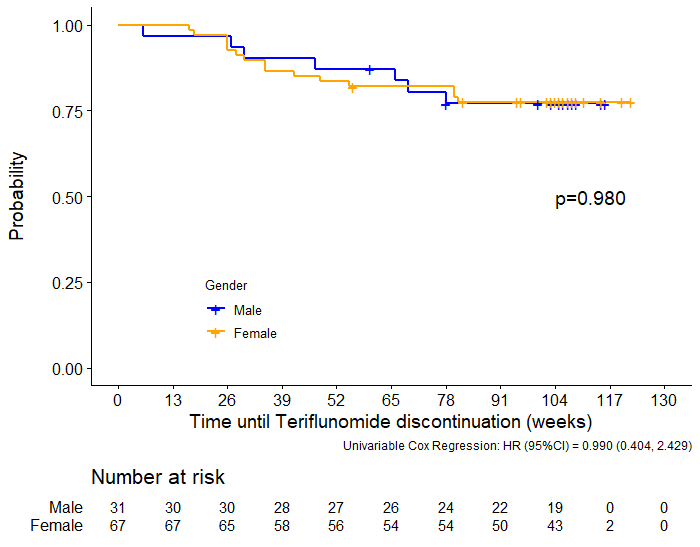

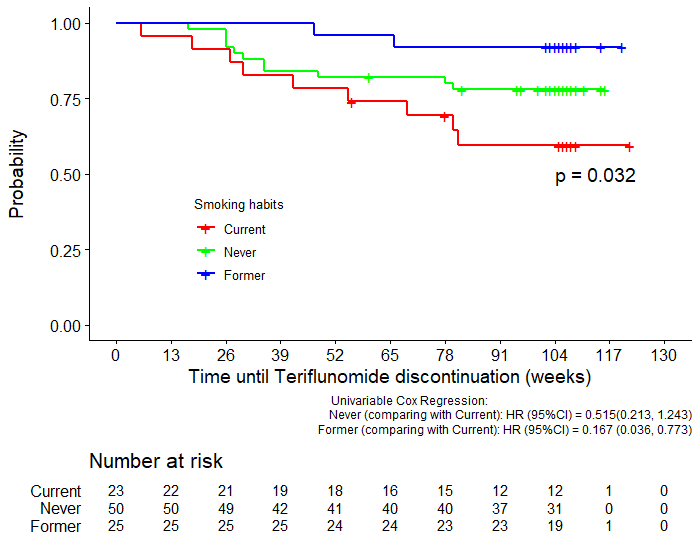

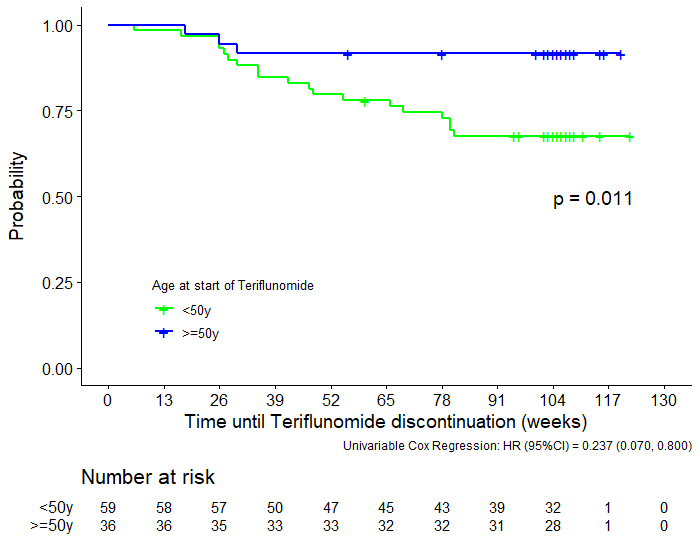

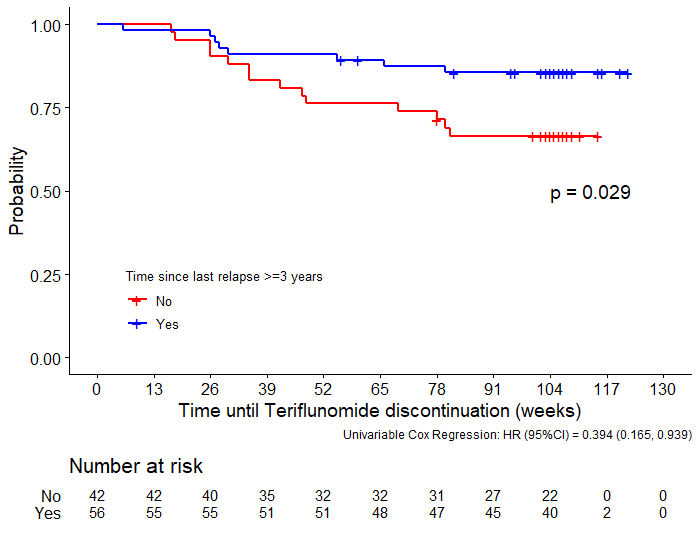


**A**

**B**

**C**

**D**

**E**

**F**

**Figure S3. Teriflunomide persistence (Kaplan-Meier estimator) in relation to previous DMTs, the time between diagnosis and drug start, time since last relapse, age, gender, and smoking habits.** Panel **A**, drug persistence of patients with or without other DMTs before teriflunomide (p<0.41); panel **B**, drug persistence of patients according to time between diagnosis and teriflunomide start (p=0.320); panel **C**, drug persistence of patients according to time since last relapse (p=0.029); panel **D**, drug persistence according to patients age at Teriflunomide start (p=0.011); panel **E**, drug persistence of patients according to their gender (p=0.980); panel **F**, drug persistence of patients according to their smoking habits (p=0.032).
